# Case Report: Intensive multidisciplinary motor-cognitive rehabilitation treatment in Gerstmann–Sträussler–Scheinker syndrome

**DOI:** 10.3389/fresc.2025.1599247

**Published:** 2026-01-23

**Authors:** A. De Laurenzis, C. Siri, L. Bandirali, L. Lucca, L. Covaia, F. Ferrari, C. Zaffina, M. Canesi

**Affiliations:** 1 Movement Disorders Rehabilitation Department, Moriggia-Pelascini Hospital, Gravedona ed Uniti, Italy; 2 Department of Brain and Behavioral Sciences, University of Pavia, Pavia, Italy

**Keywords:** Gerstmann–Sträussler–Scheinker syndrome, motor rehabilitation, neuropsychology, physiotherapy, occupational therapy, speech therapy, case report

## Abstract

Gerstmann–Sträussler–Scheinker syndrome (GSS) is a genetic, autosomal dominant prion brain disease that causes ataxia and slow cognitive decline. Herein, we describe a 34-year-old woman (S.C.B.) diagnosed with GSS (variant Pro102Leu). In 2018, she presented with gait instability without falls, early fatigue, and dizziness; after 3 years, urinary incontinence, initial insomnia and rapid-eye movement sleep behavior disorder, writing difficulties, and occasional dysphagia had appeared. S.C.B. was admitted to the Neurorehabilitation Unit due to the progressive worsening of her balance and walking ability [Timed Up and Go (TUG): not evaluated; Berg Balance Scale (BERG): 5/56]. At admission, neurological, neuropsychological, physiotherapeutic, occupational, and speech assessments were performed. She was completely dependent in basic (ADL) and instrumental activities of daily living (iADL) (ADL: 5/6; iADL: 4/8; Barthel Index: 60); furthermore, moderate cognitive decline and depressive symptoms were observed. After 4 weeks of *intensive motor-cognitive rehabilitation treatment*, an improvement was observed in all the intervention areas, leading to a global gain in autonomy (TUG: 1 min 50 s; BERG: 10/56; Barthel Index: 70). This case report highlights the importance of tailored motor-cognitive rehabilitation and how these interventions can enhance patients’ quality of life and their ability to cope with their symptoms. In conclusion, this case contributes to a broader understanding of treatment options that clinicians can propose to patients.

## 1 Introduction

Gerstmann–Sträussler–Scheinker syndrome (GSS) belongs to the group of prion diseases, which includes Creutzfeldt–Jakob disease (CJD), fatal familial insomnia (FFI), and kuru (35).

GSS is an autosomal dominant disorder caused by a mutation in the prion protein gene (*PRNP*) located on chromosome 20. Mutation P102L is the most common variant, but different mutations have been identified (such as F198S, Q217R, P105L), with each associated with less typical phenotypes of the disease (12, 13).

Spongiform degeneration is most frequently seen in patients with the P102L mutation (32). All GSS variants share widespread multicentric amyloid plaques, which are an expression of the PrP misfolded protein. A few cases of neurofibrillary tangles (NFT), found coexisting in the neocortex with amyloid plaques, have also been described and could be unique to a variant of GSS, since it is not a feature of iatrogenic or sporadic CJD (21).

GSS is a rare syndrome, with an incidence rate of 1–100 per 10 million and an age of onset ranging from 30 to 60 years. The disease follows a variable course (3.5–9.5 years) and the mean disease duration is 5 years, which is longer than classic CJD (3). The core clinical feature is the progressive cerebellar ataxia, which includes gait unsteadiness, limb ataxia with dysmetria, dysdiadochokinesis, and intention tremor. Other clinical features are pyramidal signs, Parkinsonism, and cognitive decline.

Memory impairment is usually the first indication of cognitive decline, followed by learning difficulties, slowed information processing, and reduced attention span. Cognitive decline and mood disorders seem to appear later in the course of the disease compared to motor impairment, but psychiatric disorders could be presenting symptoms, as reported in the literature (43).

There are no pathognomonic findings in standard instrumental examinations; therefore, diagnosis relies on genetic test results. MRI may show mild cerebellar atrophy and diffuse cortical atrophy, while electroencephalography reveals no specific changes, with only a small percentage of the patients (<35%) showing triphasic periodic complexes (23). There is no effective treatment for GSS and symptomatic treatment provides only partial relief.

To the best of our knowledge, this is the first description of the effects of intensive cognitive-motor rehabilitation training in a patient with GSS.

## 2 Case report

In January 2023, a 34-year-old woman (S.C.B.) who was diagnosed with GSS (variant Pro102L) in 2020 was admitted to our Neurorehabilitation Unit due to progressive worsening of balance and walking ability.

The first symptoms presented in 2018 with gait instability without falls, early fatigue, and dizziness; a brain MRI scan was performed, which only showed mild bilateral cerebellar hemisphere atrophy, leading to the diagnosis of ataxia of undetermined cause. Her symptoms progressively worsened, and a whole spine MRI scan was conducted in 2019, finding mild dorsal spinal cord hypotrophy (D4–D7) with an associated syringomyelic cavity. Motor (MEP) and somatosensory evoked potentials (SEP) showed central altered conduction abnormalities in both the upper and lower limbs. Brain-stem auditory evoked potentials (BAEPs) were normal. As of 2020, the patient has required a walker to move within her home environment. The brain and spine MRI exams were repeated in 2020, indicating the progression of the known cerebellar hypotrophy and an increase in both the length and width of the syringomyelic cavity, which extended from D4 to D11. Cerebral positron emission tomography (PET) showed cerebellar hypometabolism. Later symptoms included urinary incontinence from a neurogenic bladder (from 2021), initial insomnia and rapid-eye movement sleep behavior disorder (RBD), writing difficulties, and occasional dysphagia to a liquid bolus. Neither a familial history of GSS nor other relevant medical conditions were reported.

Considering the worsening of her symptoms and the wide range of functions affected, a holistic multidisciplinary rehabilitative approach was suggested.

S.C.B. was admitted to attend a 4-week rehabilitation inpatient program in our unit. The weekly schedule consisted of 1 h of face-to-face physiotherapy, 1 h of machine training, 1 h of speech therapy, and 1 h of group occupational therapy treatment, 5 days per week. On the sixth day, the patient had a 1 h activity in the machine room, while the seventh day was dedicated to rest. A total of 4 h per day was planned during the treatment. However, due to the patient's fatigue and attentional burden, the treatment duration was divided into shorter sessions throughout the day, while still achieving the full 4-h total.

Neurological, neuropsychological, physiotherapeutic, occupational, and speech assessments were performed in the first days after hospital admission.

The types of exercises selected were specifically chosen for the patient, without adhering to a specific rehabilitation protocol, due to the complex clinical situation involving both cerebellar symptoms and cognitive decline.

The goals, which were set with the patient, were

- greater autonomy and safety in transfers and ambulation,
- improvement of both static and dynamic balance,
- reduced fatigability,
- greater autonomy in activities of daily living (ADLs), and
- better compliance and assistance to the caregiver in the execution of activities of daily living.

The primary objective of the rehabilitation team was to formulate a Specific, Measurable, Achievable, Relevant, and Time-bound goal, in accordance with the SMART framework. Due to the severity of the symptoms and the pathology's impact on the patient's quality of life, the most significant goals were achieving better autonomy in ADLs and a safer way to manage them. Moreover, these were the most reasonable objectives to obtain for the patient, given the duration of the hospitalization, the recovery potential, and the progressive nature of the syndrome. Regarding the measurability of the chosen goals, safer management of ADLs was measured by quantitative scales, namely, Timed Up and Go (TUG), Berg Balance Scale (BERG), 6-min walking test, Barthel Index, Conley Scale, Minnesota Manual Dexterity Test, and nine-hole peg test, and a qualitative scale, namely the Parkinson's Disease Disability Scale (PDDS), to assess the patient’s initial ability level in managing postural transition and transfers and the dexterity and coordination of her upper limbs. The improvements achieved by the patient would be reflected in better performance, efficacy, and efficiency in ADLs, which the subject would then be able to replicate in her daily routine. To develop a time-based rehabilitation program, the goals were planned to be reached after 1 month of hospitalization and were measured by time-based evaluation tests.

At admission, S.C.B. was completely dependent in both ADLs and instrumental activities of daily living (iADLs). The patient entered the rehabilitation ward with her own walker and her wheelchair. She continued to use them throughout the rehabilitation period and, by the end of the hospitalization, no changes were necessary. She returned home with greater autonomy and a better understanding of how to safely use assistive devices and she was able to help her caregivers more during transfers. She was alert, cooperative, well-oriented in herself, partially oriented in space and time, and had dysarthric speech. The neurological examination showed primary position bilateral nystagmus, slow saccades, and bilateral absence of the pharyngeal reflex. Muscle tone was normal and no major deficits in active movements against gravity and resistance were observed, except for a downward drift during left arm extension. Bilateral dysdiadochokinesia and decreased finger dexterity were present, particularly on the right side. Coordination assessment revealed bilateral dysmetria during both finger-to-nose and knee-to-shin maneuvers. Hoffman's and the Babinski signs were evoked in her upper and lower limbs, respectively. Deep tendon patellar and ankle reflexes were weak bilaterally. Superficial sensitivity was normal, while hypopallesthesia and hypobathesthesia were found. The patient was able to stand and move independently with supervision. She presented with a wide walking gait and truncal ataxia with a retropulsion tendency, and therefore required a walker. Pharmacological therapy consisted of oxybutynin 5 mg q.d., gabapentin 100 mg one tablet b.i.d., lorazepam 1 mg one tablet, and cholecalciferol 10,000 UI once a month.

### 2.1 Neuropsychological evaluation

A neuropsychological assessment was conducted to evaluate the patient’s cognitive, psychological, and behavioral functioning. The results of the cognitive and emotional assessments were used to help the therapists understand how to interact with the patient by taking cognitive and/or behavioral issues into account. The test scores were adjusted by age, sex, and education and compared with Italian normative data.

During the interview, S.C.B. was alert, tidy, collaborative, and had good interaction and relationship skills. She was partially oriented in time and space, well oriented in herself, and slightly unaware of her cognitive difficulties; ideomotor slowness and moderate ataxic deficit were observed. Speech was fluent, dysarthric, and informative in content, with occasional anomies and anomic latencies detected. Verbal comprehension was preserved.

The cognitive assessment showed a moderate cognitive decline involving almost all the areas explored, except for short-term memory (reduced but within normal range) and logical reasoning (preserved) (see Table 1). In agreement with clinical observation during the interview, no anxiety was observed using the State-Trait Anxiety Inventory (STAI) scales, and mild depression on the cognitive scale was detected using the Beck Depression Inventory-II (BDI).

**Table 1:** Neuropsychological assessment.

Cognitive domain	Test	Raw score	Age-and-education-corrected score	Comment
Global cognitive assessment	Mini Mental State Examination (MMSE) (26)	22|30	22.09	Mildly compromised
	Montreal Cognitive Assessment (MoCA) (36)	13|30	11.85	Pathological
	Frontal Assessment Battery (FAB) (2)	13|18	11.7	Pathological
Language	CaGi test (11)	44|48	43.18	Borderline
	Semantic fluency	26	18.97	Pathological
	Phonemic fluency (14)	15	12.16	Pathological
Attention and executive function	Colored progressive matrices (CPM47) (4)	35|36	33	Normal
	Visual search (38)	22|60	22*	Pathological
	Trail-making test (TMT)			
	TMT A	106	113	Pathological
	TMT B	336	360	Pathological
	TMT B − A (19)	230	247	Pathological
	Stroop test			
	Error	1	2,25	Pathological
	Time (9)	35	43,75	Pathological
Memory	Digit span forward (29)	5	4,55	Borderline
	Digit span backward (29)	3	2,52	Pathological
	Corsi span forward (29)	4	3,54	Borderline
	Rey Auditory Verbal Learning Test (RAVLT)			
	Immediate recall	31|75	23,5	Pathological
	Delayed recall	6|15	3,6	Pathological
	Recognition	10|15	—	Borderline
	False positives	3		
	Intrusions (10)	9		
Visuospatial abilities	Clock drawing test (CDT) (30)	3.5|10	Cut-off 9	Pathological
Depression and anxiety	BDI-II (5)			
	Somatic/affective	7	70° pc	Normal
	Cognitive	5	92° pc	Moderate
	Total	12	80° pc	Normal
	STAI (Y1/Y2) (37)			
	STAI-Y1	46	*z* = 0.60	Normal
	STAI-Y2	51	*z* = 0.92	Normal
Functional impact	Basic activities of daily living (BADLs) (22)	5|6		Incontinence
	iADLs (24)	4|8		
				Moderate loss of autonomy in – shopping – housekeeping – drug therapy adherence – traveling

PC, percentile score.

*Score not correctable for age and education.

### 2.2 Physiotherapy assessment and intervention

An interview was conducted to collect information about the patient's difficulties in everyday life. In addition, the Conley Scale for fall prevention and for the management of hospitalized patients was administered.

Specific rating scales were used to appraise mobility, articulation in various regions, dexterity, and level of autonomy in postural transitions and transfers, balance, and walking (see Table 2).

**Table 2:** Intensive multidisciplinary motor-cognitive evaluation and rehabilitation treatment.

Treatment	Evaluation at admission	Intervention		Evaluation at discharge	Result of treatment
		Exercise	Frequency		
Speech therapy	PVD (17) Breathing: 3/4 Phonation: 3/4 Facial musculature: 2/4 Diadochokinesis: 1/4 Reflexes: 2/4 Articulation: 2/4 Intelligibility: 2/4 Prosody: 2/4 MPT: 11 s Average loudness: 90–92 dB	– Single and group sessions to improve the strength and coordination of her tongue, cheeks, lips, and suprahyoid muscles with specific swallowing exercises; – Effective intervention strategies, compensations, and behavioral strategies during meals; – Articulatory placement cues; mirror therapy; work in a couple through conversations, guided dialogs, and readings to improve speech abilities.	5 days/week	PVD (18) Breathing: 4/4 Phonation: 4/4 Facial musculature: 2/4 Diadochokinesis: 1/4 Reflexes: 3/4 Articulation: 3/4 Intelligibility: 4/4 Prosody: 3/4 MPT: 16 s Average loudness: 99–98 dB	Improvement in articulatory competence and awareness, with more precise and harmonious speech, although bradylalia persisted; greater awareness of her swallowing skills.
				– GIRBAS scale (15) Global score: 1	
	GIRBAS scale (15) Global score: 2				
	CBA: 2.5/28 (25)			– RSST: 7 respiratory acts/30 s (cut-off 3) (31)	
	DRS: 0/18.5 (1)				
	– RSST: 6 respiratory acts/30 s (cut-off 3) (31)				
Physiotherapy	Conley scale: 5/10 (20)	– Exercises to strengthen axial and limb muscles; – Training in postural transitions and transfers, walking and exertion; – Machines training (treadmill: about 1.3 km/h; cycle ergometer: 10 min; stabilometric treadmill).	Front-to-front intervention (5 days/week)	6MWT: 50 m	Improvement in static and dynamic balance and in the quality of walking.
			Machine training (6 days per week)		
				BBS: 10/56	
	6-minute walking test (6MWT): 30 m (42)				
	BBS: 5/56 (6)			TUG: 1 min50 s	
	TUG: NV			Barthel Index: 70	
	Barthel Index: 60				
Occupational therapy	Self-assessment Parkinson's Disease Disability Scale (SPDDS): 85/125 (8)	– Exercises for digital dexterity enhancement and praxis deficit recovery; – Basic exercises to improve autonomous management of ADLs (fastening buttons, folding clothes, pouring drinks from a bottle, and cutting food with cutlery, facilitating meal management); – Writing training; – Exercises with orthopedic paste to improve strength and precision of fine gestures and working on maintaining correct joint ranges in hands and fingers.	5 days/week	SPDDS: 84/125	Improvement in digital dexterity bilaterally and bimanual coordination and greater accuracy in ADLs; improvement in writing ability.
	Minnesota Manual Dexterity Test (MMDT) (16, 39, 41) P1: 460.87s P2: 355.24s T1: 133.34s T2: 128.62s T3: 127.07s T4: 102.16s			MMDT: P1: 418.19″ P2: 372.36″ T1: 124.91″ T2: 116.35″ T3: 124.06″ T4: 109.09″	
				9HPT RH: 102.64″ LH: 88.70″	
	Nine-hole peg test (9HPT) (17, 33, 34) RH: NV LH: 101.03s				

MPT, maximum phonation time.

The assessment showed a deficit in static and dynamic balance, increased fatigue, and difficulty in walking and handling the aid, requiring supervision by a caregiver and a walker. Walking was characterized by an ataxic gait.

No joint limitations emerged during passive mobilization. Generalized hyposthenia was found predominantly at the axial level and in the pelvic stabilizers. The patient required minimal assistance during postural transitions and walking transfers. She was able to maintain a static sitting position without support despite poor trunk control in dynamic trials. She could stand up with minimal assistance, but she required anterior support to maintain the position.

After the assessments, S.B.C. started physiotherapy rehabilitation training with specific exercises (Table 2) designed to increase autonomy and safety during transfers and ambulation, improve static and dynamic balance, and reduce subjective fatigability.

At the end of the intensive rehabilitation training, these goals were achieved, as shown by the improvements in the mobility tests (see Table 2).

The patient was instructed on strategies and exercises to be continued at home.

### 2.3 Speech and swallow evaluation and intervention

The speech-language pathology (SLP) assessment included an interview with the patient that evaluated communication and swallowing status, a review of motor and cognitive status, and standardized measures of specific aspects of voice, speech, spoken language, and swallowing function, including observations and the analysis of work samples.

Slurred speech emerged during the interview and the patient reported difficulty in making herself understood by other people, but this never progressed to SLP. The content and form of the sentences were simple and almost childlike. Comprehension was good despite the hearing difficulties.

The perceptual evaluation of her dysarthria, assessed using the “*Profilo di Valutazione della Disartria* (PVD)” and “GIRBAS scale”, showed mild dysarthric speech with a deficit in pneumo-phonic coordination, harsh vocal attack, rhinolalia, unstable and asthenic voice, severe hypotonia of the orofacial muscles, prognathism, improper tongue posture, and inaccurate articulation of complex and vocalic sounds, leading to disprosodic and bradilalic spoken language.

During the interview, S.B.C. reported occasional dysphagia and a swallowing assessment was performed using the Clinical Bedside Assessment (CBA), Dysphagia Risk Score scale (DRS), and Repetitive Saliva Swallow Test (RSST), which showed mild dysphagia and initial difficulty in swallowing liquids and solids (see Table 2).

Specific exercises were therefore proposed to improve the patient’s difficulties in swallowing and speech ability (see Table 2).

At discharge, the final assessment revealed an increase in articulatory competence, as her speech was more precise and harmonious, although bradylalia persisted.

Furthermore, the patient gained greater awareness of her swallowing skills and was able to safely eat solid and liquid boluses, a significant increase in her quality of life.

### 2.4 Occupational therapy evaluation and intervention

S.C.B. lives in a detached house with her husband and her young daughter. She is completely dependent in all the basic and complex ADLs. She also reported extreme difficulty in writing.

Specific tests to determine her perceived overall level of autonomy in ADL management and manual dexterity performance in different aspects were administered (see Table 2).

The assessments showed a severe decrease in hand dexterity and bimanual coordination, severe motor impairment in digital dexterity in her left hand (her right hand could not be assessed due to significant apraxia), dysmetria, global bradykinesia, and a slowed and ataxic gait.

The training consisted of specific exercises to address the main difficulties (see Table 2).

At the final evaluation, an improvement in digital dexterity bilaterally in bimanual coordination and greater control accuracy during meaningful activities was recorded; there was also excellent improvement in hand gestures in both hands. The patient’s writing improved, as it was more comprehensible and had more defined strokes in the final assessment. Finally, the patient maintained her self-perception and motivation to participate in ADLs.

The patient was instructed on strategies and exercises to be continued at home.

At discharge, the patient and her family were provided with a booklet containing suggested exercises, illustrated with both text and images. They received instructions on necessary home environment adaptations and were given a list of telephone numbers and email addresses to contact if needed.

## 3 Discussion

We present the case of a patient with GSS who attended inpatient intensive motor-cognitive rehabilitation training. Several structured rehabilitative approaches have been described and found effective for hereditary ataxias (7, 27, 28), improving mobility function, balance, and ataxia. However, to the best of our knowledge, this is the first description of rehabilitative training and its results in a patient with GSS.

In this patient with GSS, the clinical features, symptom progression, and neuroradiological findings were in line with the literature (40).

In line with the *intensive multidisciplinary motor-cognitive rehabilitation treatment* approach, the patient was carefully assessed and the intervention was adapted to her needs, despite this treatment not being designed for GSS specifically, as the approach is very adaptable. Since only a few patients with GSS have been referred for intensive rehabilitation treatment, we believe that this case may help clinicians consider *intensive multidisciplinary motor-cognitive rehabilitation treatment* as a feasible and useful tool for treating these patients.

The neuropsychological assessments painted a picture of the patient’s cognitive difficulties, allowing the therapists to adapt the communication and the way they delivered the training. The team was trained to consider the cognitive ability of the patient and to offer explanations that the patient would understand, thus helping maintain concentration (e.g., splitting one session into two to three shorter ones or frequently giving the patient a break and engaging in simple conversation). Therefore, despite some degree of cognitive dysfunction, the patient was able to perform and participate in the intervention thanks to the care provided by the team. As a result, an improvement was observed in all the intervention areas. Speech therapy was effective on her dysphagia, physiotherapy led to better walking and balance abilities, and occupational therapy allowed the patient to achieve better bimanual coordination and greater control of gestures. Overall, the 4-week *intensive motor-cognitive rehabilitation treatment* led to a global improvement in autonomy, with a positive impact on ADLs, such as improved self-management (mainly due to better balance, dexterity, and swallowing) within the hospital setting. At the end of the rehabilitation process, all the physiotherapy tests showed a slight improvement despite remaining pathological. This was not unexpected, considering the nature and severity of the pathology, which did not allow for the same outcomes as in less compromised patients. However, the approach focused on physiotherapy treatment, particularly on core postural control, which resulted in an improvement in the safety of postural transition and transfers in the domestic environment. From the occupational therapy point of view, the tests showed maintenance of a significant impairment in upper limb gestures (in gross and fine motor skills), but good improvement in terms of the time needed to finish the requested task. In terms of ADL autonomy progress, the patient went home with better fine motor control, which helped her in writing and small hygiene tasks and, during meal management, cutting and preparing her own food.

We acknowledge that the use of scales originally developed for other movement disorders may be questionable, but, as there are no instruments specifically developed for GSS, and since we used them for test-retest aims, we believe it may be an acceptable solution.

Unfortunately, we did not have the opportunity to perform a follow-up visit, which could have provided insight into the persistence of the improvements achieved, which is clinically important information.

However, it is reasonable to assume that, given this patient improved in many aspects after rehabilitation treatment was started at least 3 years after her diagnosis, an early intervention would be more effective in terms of improvement in quality of life.

We are aware that the generalizability of a single case is poor, but, considering that patients with GSS are rare, and treatment options are scarce, we believe it is important to highlight whatever may help patients to better cope with their symptoms.

In conclusion, since GSS is a relentlessly progressive disease, we believe that being able to obtain sensible results with a rehabilitative treatment is an important step to improve these patients’ quality of life. Furthermore, describing cases such as this increases our knowledge about treatment possibilities that clinicians can propose to patients. There is a lack of studies on the efficacy of rehabilitative training in people with GSS and randomized studies with longitudinal follow-up should be conducted to further explore rehabilitative options and confirm the efficacy of the intervention in this patient population.

## Data Availability

The original contributions presented in the study are included in the article/Supplementary Material, further inquiries can be directed to the corresponding author.
